# Association Between Food Insecurity and Serious Psychological Distress Among Hispanic Adults Living in Poverty

**DOI:** 10.5888/pcd12.150334

**Published:** 2015-11-25

**Authors:** Benjamin J. Becerra, Reacheal Connie Sis-Medina, Alexa Reyes, Monideepa B. Becerra

**Affiliations:** Author Affiliations: Reacheal Connie Sis-Medina, Alexa Reyes, Monideepa B. Becerra, Department of Health Science and Human Ecology, California State University, San Bernardino, California.

## Abstract

**Introduction:**

Food insecurity has been associated with negative health outcomes, but the relationship between psychological distress and food insecurity among ethnic minorities has not been extensively examined in the literature. The goal of this study was to evaluate whether low food security and very low food security were significantly associated with past month serious psychological distress (SPD) among Hispanic adults living in poverty.

**Methods:**

We studied 10,966 Hispanic respondents to the California Health Interview Survey for 2007, 2009, and 2011–2012 whose income was below 200% of the federal poverty level. The relationship between food insecurity and SPD was evaluated by using survey-weighted univariate and logistic regression analyses.

**Results:**

Nearly 30% of the study population had low food security and 13% had very low food security. Low food security and very low food security were associated with 1.99 and 4.43 odds of past month SPD, respectively, and perceived low neighborhood safety was related to 1.47 odds of past month SPD.

**Conclusions:**

We found that food insecurity was prevalent among Hispanic people living in poverty and was significantly associated with past month SPD. These results demonstrate the need for further targeted public health efforts, such as community gardens led by *promotores,* faith-based initiatives, and initiatives to reduce barriers to participation in food-assistance programs.

## Introduction

The US Department of Agriculture (USDA) defines food security as “access by all people at all times to enough food for an active, healthy life,” and it is a critical aspect to ensuring population health. The USDA also notes that hunger is a “. . . prolonged, involuntary lack of food that results in discomfort, illness, weakness, or pain that goes beyond the usual uneasy sensation” and is often termed very low food security (1). Furthermore, Hispanics are more likely to report high food insecurity than non-Hispanic whites (24% vs 11%) (2).

Food insecurity is significantly associated with worse health outcomes. For instance, low household food security results in reduced consumption of healthy food among Hispanic children (3,4) and in substance abuse among Hispanic women (5). In a qualitative study, Quandt et al examined the negative psychological effects of food insecurity, especially those related to fear due to lack of immigration documents, embarrassment, and guilt (6). Such results indicate the need for further quantitative assessment to investigate the role of food insecurity in the psychological health of the US Hispanic population.

Understanding how such a factor can affect the mental health of Hispanics is imperative, given the shift in the nation’s health care priorities from specialty care to integrated care (7). We evaluated the relationship between food security status and serious psychological distress (SPD) in the past month among Hispanics living in poverty, using the largest state health population-based survey in the United States.

## Methods

We conducted a secondary analysis of the public use, adult portion of the California Health Interview Survey (CHIS), 2007, 2009, and 2011–2012. CHIS, a biennial survey, is conducted in several languages, including Spanish. CHIS uses a random-digit–dial system inclusive of both landline and cellular telephones to select participants from all Californians, excluding homeless, incarcerated, and institutionalized people and those living in group homes. Response rates of CHIS are comparable with those of other state health surveys, such as the Behavioral Risk Factor Surveillance System; further details on CHIS can be found elsewhere (8). We included 10,966 self-reported Hispanics aged 18 years or older who reported living below 200% of the federal poverty level (FPL).

The outcome variable in this study was SPD in the past month, measured in CHIS as scoring 13 or higher in the Kessler-6 scale, a validated instrument (9,10). The scale comprises 6 questions on anxiety and depressive symptoms; scores range from 6, indicating no distress, to 30, indicating severe distress (11,12).

The primary exposure variable in this study was household food security status. CHIS provides 3 variables on poverty and food security: food secure, food insecure without hunger, and food insecure with hunger. People living below 200% of the FPL (or those who selected “unknown”) were asked the following questions: 1) “The food that (I/we) bought just didn’t last, and (I/we) didn’t have money to get more,” 2) “(I/We) couldn’t afford to eat balanced meals,” 3) “In the last 12 months, did you or other adults in your household ever cut the size of your meals or skip meals because there wasn’t enough money for food?” 4) “How often did this happen? Almost every month, some months but not every month, or only in 1 or 2 months?” 5) “In the last 12 months, did you ever eat less than you felt you should because there wasn’t enough money to buy food?” and 6) “In the last 12 months, were you ever hungry but didn’t eat because you couldn’t afford enough food?” The last question denoted food insecurity with hunger in the CHIS. In this study, we used the CHIS-provided food security variable and defined food insecurity without hunger as low food security and food insecurity with hunger as very low food security, per USDA guidelines (1).

Control variables were age (18–24 y, 25–44 y, 45–64 y, and 65 or more years); sex (male, female); marital status (married, not married); employment status (employed, unemployed); education level (high school diploma or less, some college [vocational or Associate degree], Bachelor degree or higher), and country of birth (US-born, foreign-born). Risk behaviors (smoking and/or binge drinking in the past year), presence of at least one chronic disease, and neighborhood safety were used as control variables. Smoking behavior was defined as current smoker versus not a current smoker, and binge drinking was defined as consuming 5 or more drinks for men and 4 or more drinks for women on one occasion. Chronic disease was classified as having at least one of the following conditions: heart disease, congestive heart failure, hypertension, type 2 diabetes, or asthma. CHIS provided a neighborhood safety variable based on the question “Do you feel safe in your neighborhood?” Response options were all of the time, most of the time, some of the time, and none of the time. We dichotomized responses as some or none of the time versus all or most of the time. We included participation in the Supplemental Nutrition Assistant Program (SNAP) as a control variable, identified via the question of “Are you receiving Food Stamp benefits, also known as CalFresh?”

All statistical analyses were survey weighted and conducted using SAS version 9.4 (SAS Institute, Inc). To determine the distribution of SPD by each population characteristic, we used design-based *F* values for bivariate analyses. Next, we used multivariable logistic regression analyses to evaluate the association between food insecurity and SPD, after adjusting for control variables and survey year. We also evaluated the contribution of each set of variables on past month SPD by using the c-statistic from the area under the curve/receiver operating characteristic (AUC/ROC). An ɑ level of .05 was used to determine significance. The study was approved by the institutional review board of California State University, San Bernardino.

## Results

Nearly 30% of the study population reported having low food security (n = 1,124,998), and approximately 13% had very low food security (n = 507,955) (Table 1). Approximately 15% reported participating in SNAP. An average of 5%, or 192,741 Hispanic adults, reported SPD. The highest proportion of our study population was aged 25 to 44 years (48%), was female (52%), was not married (54%), had a high school degree or less (81%), was employed (59%), and was foreign-born (72%). Nearly 28% of the population reported engaging in at least one risk behavior (smoking or binge drinking) and having at least one chronic disease (33%); approximately 23% reported their neighborhood to be safe some or none of the time.

**Table 1:** Study Population (n = 10,966) Characteristics, California Health Interview Survey, 2007, 2009, and 2011–2012^a^

Characteristic	Average Annual Population Estimate (N = 3,776,299)	n (Weighted %)
**Age, y**		
18–24	749,771	1,683 (19.9)
25–44	1,824,038	4,601 (48.3)
45–64	919,453	3,237 (24.3)
≥65	283,037	1,445 (7.5)
**Sex**		
Male	1,822,516	4,187 (48.3)
Female	1,953,783	6,779 (51.7)
**Marital status**		
Not married	2,022,551	6,000 (53.6)
Married	1,753,748	4,966 (46.4)
**Education level**		
High school diploma or less	3,053,612	8,673 (80.9)
Some college/vocational/associate	552,860	1,684 (14.6)
Bachelor or higher degree	169,827	609 (4.5)
**Employment status**		
Employed	2,214,326	5,793 (58.6)
Unemployed	1,561,974	5,173 (41.4)
**Country of birth**		
Foreign-born	2,710,403	7,932 (71.8)
US-born	1,065,896	3,034 (28.2)
**Risk behavior^b^**		
Smoke and binge drink	240,647	561 (6.4)
Smoke or binge drink	1,043,660	2,613 (27.6)
None	2,491,991	7,792 (66.0)
**Chronic disease**		
No	2,521,730	6,748 (66.8)
Yes	1,254,570	4,218 (33.2)
**Neighborhood safety**		
Safe only some or none of the time	855,994	2,297 (22.7)
Safe all or most of the time	2,920,306	8,669 (77.3)
**Food security status^c^**		
Food secure	2,143,347	6,310 (56.8)
Low food security	1,124,998	3,230 (29.8)
Very low food security	507,955	1,426 (13.4)
**SNAP participation**		
Yes	562,292	1,838 (14.9)
No	3,207,250	9,100 (85.1)
**Past month SPD**		
Yes	192,741	685 (5.1)
No	3,583,558	10,281 (94.9)
**Survey year**		
2007	1,072,117	3,244 (28.4)
2009	1,328,113	3,645 (35.2)
2011–2012	1,376,069	4,077 (36.4)

Abbreviations: SNAP, Supplemental Nutrition Assistance Program; SPD, serious psychological distress.

a Values may not sum to total value for N due to missing data or rounding.

b Binge drinking was defined as consuming 5 or more drinks for men and 4 or more drinks for women on one occasion.

c We defined food insecurity without hunger as low food security and food insecurity with hunger as very low food security.

Table 2 demonstrates the prevalence of SPD by population characteristics. With the exception of education, country of birth, and risk behaviors, significant differences were noted for each variable. For example, an increasing prevalence of SPD was noted with worsening food security status; the highest percentage was found among respondents with very low food security (13%), and the lowest was found among those who reported being food secure (3%). A higher prevalence of SPD was found among respondents with at least one chronic disease than among those with no chronic disease (8% vs 4%); it was also higher among those reporting low neighborhood safety than among those who reported their neighborhood as safe all or most of the time (8% vs 4%). Respondents who were unemployed, not married, aged 45 to 64 years, and female had a higher prevalence of SPD than their counterparts. Respondents who participated in SNAP reported a higher prevalence of past month SPD (8%) than those who did not participate in SNAP (5%).

**Table 2:** Prevalence of Past Month SPD, California Health Interview Survey, 2007, 2009, and 2011–2012

Characteristic	Past Month SPD, Weighted % (95% CI)	*P* Value
**Food security status^a^**		
Food secure	2.67 (2.09–3.25)	<.001
Low food security	6.00 (4.59–7.41)	
Very low food security	13.40 (11.09–15.71)	
**SNAP participation**		
Yes	8.37 (6.19–10.56)	<.001
No	4.54 (3.95–5.13)	
**Age, y**		
18–24	3.16 (2.03–4.30)	<.001
25–44	4.29 (3.49–5.09)	
45–64	8.49 (6.71–10.26)	
≥65	4.50 (3.19–5.81)	
**Sex**		
Male	4.20 (3.26–5.13)	.005
Female	5.95 (5.16–6.74)	
**Marital status**		
Not married	5.91 (5.05–6.77)	.003
Married	4.17 (3.33–5.02)	
**Education level**		
High school diploma or less	5.28 (4.53–6.03)	.35
Some college/vocational/associate	4.51 (3.14–5.88)	
Bachelor or higher degree	3.84 (1.88–5.80)	
**Employment status**		
Employed	4.04 (3.28–4.80)	<.001
Unemployed	6.61 (5.44–7.78)	
**Country of birth**		
Foreign-born	5.34 (4.55–6.14)	.20
US-born	4.50 (3.48–5.51)	
**Risk behavior^b^**		
Smoke and binge drink	7.27 (3.91–10.64)	.054
Smoke or binge drink	5.93 (4.43–7.42)	
None	4.55 (3.94–5.16)	
**Chronic disease**		
No	3.59 (2.84–4.34)	<.001
Yes	8.15 (6.92–9.38)	
**Neighborhood safety**		
Safe only some or none of the time	7.66 (5.98–9.33)	<.001
Safe all or most of the time	4.36 (3.72–4.99)	
**Survey year**		
2007	5.64 (4.61–6.68)	.007
2009	3.74 (2.80–4.68)	
2011–2012	6.00 (4.71–7.29)	

Abbreviations: CI, confidence interval; SNAP, Supplemental Nutrition Assistance Program; SPD, serious psychological distress.

a We defined food insecurity without hunger as low food security and food insecurity with hunger as very low food security.

b Binge drinking was defined as consuming 5 or more drinks for men and 4 or more drinks for women on one occasion.

After adjusting for control variables, in addition to survey year, Hispanic adults in poverty who reported very low food security had 4.43 greater odds of reporting SPD than those who were food secure (Table 3). Low food security was also associated with increased likelihood of SPD (adjusted odds ratio [OR] = 1.99, 95% confidence interval [CI] = 1.44–2.76). SNAP participants reported 1.61 greater odds of past month SPD (95% CI = 1.18, 2.21) than nonparticipants. Assessment of food security status and SNAP participation did not yield significant results.

**Table 3:** Association Between Food Security Status and Past Month SPD Among Hispanic Adults in Poverty, California Health Interview Survey, 2007, 2009, and 2011–2012

Characteristic	Adjusted OR (95% CI)	*P* Value
**Food security status^a^**		
Food secure	1 [Reference]	
Low food security	1.99 (1.44–2.76)	<.001
Very low food security	4.43 (3.14–6.24)	<.001
**SNAP Participation**		
No	1 [Reference]	
Yes	1.61 (1.18–2.21)	.003
**Age, y**		
18–24	1 [Reference]	
25–44	1.16 (0.72–1.88)	.54
45–64	2.27 (1.39–3.71)	.001
≥65	1.16 (0.71–1.89)	.55
**Sex**		
Male	1 [Reference]	
Female	1.42 (1.05–1.91)	.02
**Marital status**		
Married	1 [Reference]	
Not married	1.57 (1.20–2.06)	.001
**Education level**		
Bachelor or higher degree	1 [Reference]	
High school diploma or less	1.17 (0.68–1.99)	.57
Some college/vocational/associate degree	1.24 (0.68–2.25)	.49
**Employment status**		
Employed	1 [Reference]	
Unemployed	1.65 (1.22–2.24)	.001
**Country of birth**		
US-born	1 [Reference]	
Foreign-born	1.25 (0.89–1.77)	.20
**Risk behavior^b^**		
None	1 [Reference]	
Smoke and binge drink	2.33 (1.35–4.05)	.003
Smoke or binge drink	1.53 (1.09–2.15)	.01
**Chronic disease**		
No	1 [Reference]	
Yes	1.89 (1.35–2.65)	<.001
**Neighborhood safety**		
Safe all or most of the time	1 [Reference]	
Safe only some or none of the time	1.47 (1.11–1.96)	.008
**Survey year**		
2007	1 [Reference]	
2009	0.54 (0.38–0.75)	<.001
2011–2012	0.81 (0.60–1.08)	.15

Abbreviations: CI, confidence interval; OR, odds ratio; SNAP, Supplemental Nutrition Assistance Program; SPD, serious psychological distress.

a We defined food insecurity without hunger as low food security and food insecurity with hunger as very low food security.

b Binge drinking was defined as consuming 5 or more drinks for men and 4 or more drinks for women on one occasion.

Sociodemographic factors significantly associated with past month SPD were being aged 45 to 64 years (adjusted OR = 2.27, 95% CI = 1.39–3.71), female (adjusted OR = 1.42, 95% CI = 1.05–1.91), not being married (adjusted OR = 1.57, 95% CI = 1.20–2.06), and being unemployed (adjusted OR = 1.65, 95% CI = 1.22–2.24). A dose-dependent association was noted for risk behaviors; engaging in at least one risk behavior (smoking or binge drinking) was associated with a 1.53 greater odds (95% CI = 1.09–2.15) of past month SPD, and engaging in both of those behaviors was associated with a 2.33 greater odds (95% CI = 1.35–4.05) of past month SPD. Increased odds of past month SPD was also associated with reporting a neighborhood to be safe some or none of the time (adjusted OR = 1.47, 95% CI = 1.11–1.96), compared with those who reported it to be safe all or most of the time. Past month SPD was also associated with having at least one chronic disease (adjusted OR = 1.89, 95% CI = 1.35–2.65), compared with respondents who reported none. AUC/ROC analysis demonstrated that food security status had the highest relative contribution (change in c-statistic, 0.051) to past month SPD in our study population, after accounting for education level and employment status (Table 4).

**Table 4:** Contribution of Each Set of Variables to Model for Past Month SPD, California Health Interview Survey, 2007, 2009, and 2011–2012

Variable^a^	c-Statistic	Change
None	0.5	—
+ Year	0.536	0.036
+ Age, sex, marital status	0.646	0.11
+ Education, employment	0.662	0.016
+ SNAP participation	0.667	0.005
+ Risk behaviors, chronic disease, country of birth	0.696	0.029
+ Neighborhood safety	0.708	0.012
+ Food security status	0.759	0.051

Abbreviations: —, not applicable; SNAP, Supplemental Nutrition Assistance Program; SPD, serious psychological distress.

a Analyses conducted using area under the curve/receiver operating characteristic (AUC/ROC) analysis and were adjusted for education level and employment status.

## Discussion

The American Dietetic Association states that addressing food insecurity and hunger in the United States is imperative (13). Food insecurity and hunger are public health issues that result in negative health outcomes, including obesity (14), poor dietary intake (15), and mental illness and gastrointestinal infections (16); the role of food insecurity on the psychological wellbeing of Hispanics is limited, so we addressed such a gap in the literature.

We found that nearly 30% of Hispanic adults living in poverty reported low food security, and approximately 13% reported very low food security; both rates are substantially higher than national rates. Our results also indicated that Hispanics with very low food security had 4.4 higher odds of past month SPD than those who were food secure, which demonstrates the substantial burden of hunger in the population, even after accounting for SNAP participation. These results indicate the need for public health interventions (in addition to SNAP) to alleviate the prevalence of very food insecure households among one of the largest US minority populations.

Although government programs such as SNAP may mitigate such a burden, the residential requirements may be a barrier, especially for people with undocumented status. Federal reports indicate that Hispanics are 4 times more likely than whites not to apply for food stamps, despite being eligible, often citing a lack of knowledge as a barrier (17). More public health efforts, such as outreach to educate on eligibility, are needed to reach vulnerable populations to alleviate the health burden associated with food insecurity and hunger. Such an outreach program would provide an opportunity to integrate community health workers, or *promotores de salud*, into health promotion programs, such as community gardens (18), to improve health outcomes of vulnerable populations.

Stigma, cost of application, and travel time are also barriers to participation in government programs (17). Such factors may be attributable to the significant prevalence of low and very low food security found in our study, and government programs are critical for public health efforts to improve food security in the Hispanic population. Another opportunity exists in the Obama administration’s call for integration of faith-based organizations in improving health outcomes of Americans (19). Such organizations have been involved in providing opportunities for preventive care, routine screening, and health education opportunities and can also help with outreach to food insecure households (20).

The USDA makes a distinction between food insecurity and hunger, the former being a measure of socioeconomic status and the latter being a physiological state (1). In alignment with such a distinction, our results illustrate the burden of very low food security (which includes hunger) on the mental health status of Hispanic adults and not just with food insecurity without hunger. A similar association between food insecurity, hunger, and obesity among minority women was noted by a previous study (14), cumulatively highlighting the need for researchers to expand beyond food insecurity as a predictor for poor health status among vulnerable populations and address the importance of hunger.

Our results indicated that being female, not being married, being unemployed, having at least one chronic disease, and engaging in risk behaviors (smoking and/or binge drinking) are associated with poor mental health status, and such results are consistent with other studies’ findings; however, the association between perceived poor neighborhood safety and SPD among Hispanics in poverty warrants further discussion. Researchers have found an association between low social cohesion and high rates of depression and smoking (21), between low neighborhood safety and low physical activity (21,22), and between poor neighborhood conditions, such as boarded-up housing, and increased rates of chronic diseases, homicide, suicide, and death (23). Our results indicated that perceived neighborhood safety plays a substantial role on past month SPD, further demonstrating the importance of neighborhood contextual factors in health outcomes, including mental health, and the imperative need for community-based interventions to improve neighborhood conditions to ensure equity in health outcomes. The several significant factors associated with past month SPD found in our study, in addition to the relationship with food insecurity, further highlight at-risk groups in an already stigmatized population and thus the imperative need for targeted public health initiatives.

Our study has limitations. The potential for selection and self-report biases exists in any study that uses survey data. The CHIS survey, however, is conducted in Spanish and, therefore, may not necessarily limit respondents with low English language proficiency; this factor may in turn have provided a higher rate of inclusion of Hispanics in California. Some variables, such as marital status and employment status, were dichotomized, so we could not assess a more thorough relationship between such characteristics and past month SPD. CHIS is conducted in California, so the results may not be generalizable to other states. Our results do provide the foundation for further research on the role of food insecurity in health conditions.

The results of this study have significant implications for public health practice. Given the high prevalence of low and very low food security among the adult Hispanic population living in poverty, heightened efforts to improve food security are critical, especially in light of the negative health effects noted in the literature and our study. The results associated with poor neighborhood safety also demonstrate the social context of health and the need for public health efforts in equity in social determinants to improve population health.
